# Spotlight on therapeutic efficiency of mesenchymal stem cells in viral infections with a focus on COVID-19

**DOI:** 10.1186/s13287-022-02944-7

**Published:** 2022-06-17

**Authors:** Saman Yasamineh, Hesam Ghafouri Kalajahi, Pooneh Yasamineh, Omid Gholizadeh, Hamed Rahmani Youshanlouei, Saeed Karimi Matloub, Masoud Mozafari, Elham Jokar, Yalda Yazdani, Mehdi Dadashpour

**Affiliations:** 1 Department of Medical Microbiology, Faculty of Medicine, Tabriz University of Medical Sciences, Tabriz, Iran; 2 Young Researchers and Elite Club, Tabriz Branch, Islamic Azad University, Tabriz, Iran; 3 Department of Biotechnology, Institute of Science, Uskudar University, Istanbul, Turkey; 4 Department of Bacteriology and Virology, Faculty of Medicine, Tabriz University of Medical Sciences, Tabriz, Iran; 5 Department of Internal Medicine, Faculty of Medicine, Tabriz University of Medical Sciences, Tabriz, Iran; 6 Students Research Committee, Qom University of Medical Sciences, Qom, Iran; 7 Cardiovascular Pharmacology Research Lab, Department of Pharmacology, School of Medicine, Shiraz University of Medical Sciences, Shiraz, Iran; 8 Department of Medical Chemistry, School of Pharmacy, Ahvaz Jundishapur University of Medical Sciences, Ahvaz, Iran; 9 Immunology Research Center, Tabriz University of Medical Sciences, Tabriz, Iran; 10 Cancer Research Center, Semnan University of Medical Sciences, Semnan, Iran

**Keywords:** Stem cells therapy, Mesenchymal stem cells, SARS-CoV-2, COVID-19

## Abstract

The SARS-COV-2 virus has infected the world at a very high rate by causing COVID-19 disease. Nearly 507 million individuals have been infected with this virus, with approximately 1.2% of these patients being dead, indicating that this virus has been out of control in many countries. While researchers are investigating how to develop efficient drugs and vaccines versus the COVID-19 pandemic, new superseded treatments have the potential to reduce mortality. The recent application of mesenchymal stem cells (MSCs) in a subgroup of COVID-19 patients with acute respiratory distress has created potential benefits as supportive therapy for this viral contagion in patients with acute conditions and aged patients with severe pneumonia. Consequently, within this overview, we discuss the role and therapeutic potential of MSCs and the challenges ahead in using them to treat viral infections, with highlighting on COVID-19 infection.

**Graphical Abstract:**

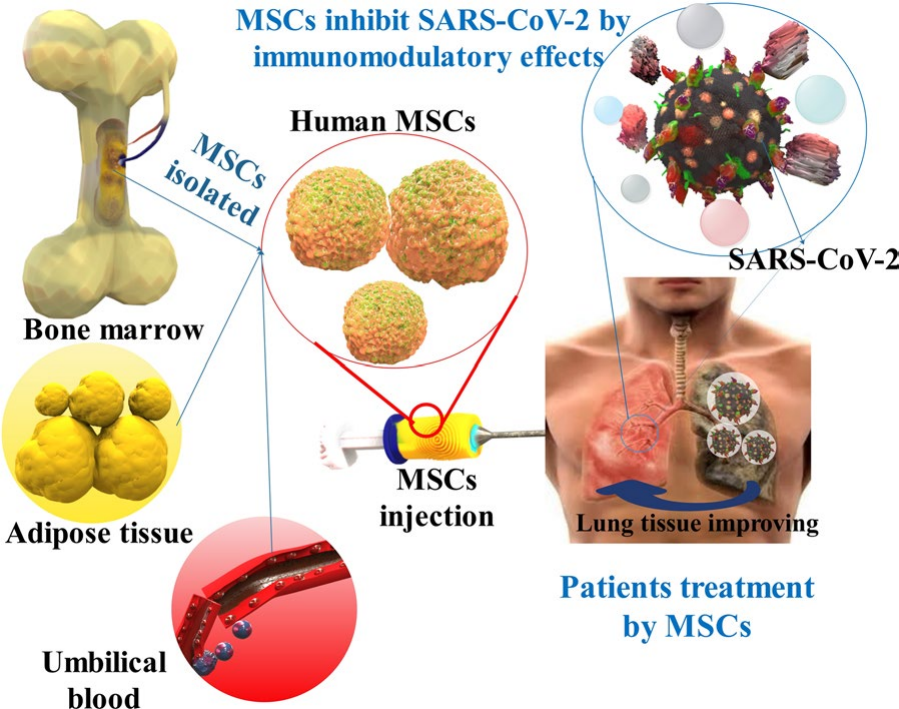

## Introduction

**Fig. 1:**
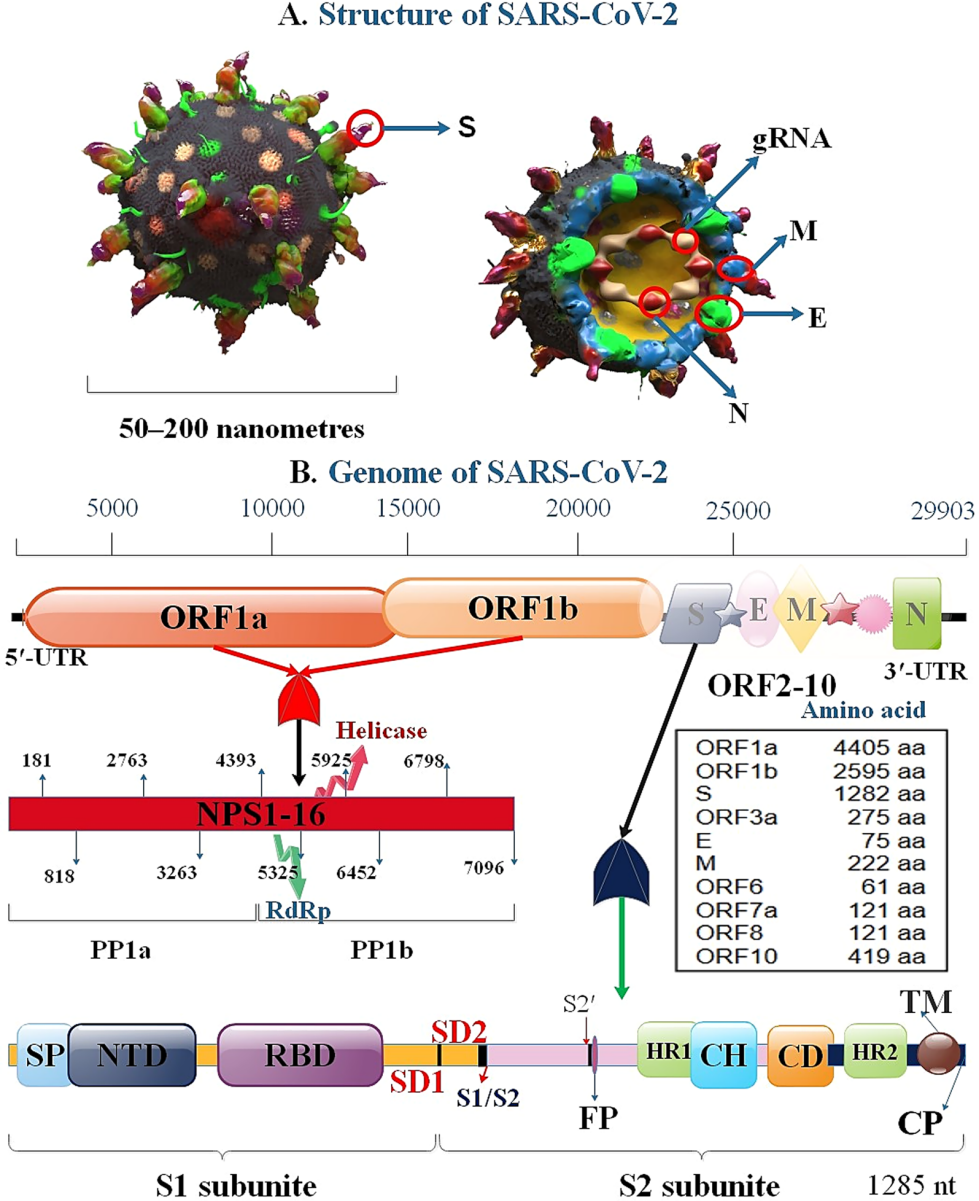
3D-graphical illustrations of the structural proteins of SARS-CoV-2 (**A**) and genome (**B**). S protein genetic site contained SP (single peptide), CP (cytoplasmic tail), FP (fusion peptide), CD (connector domain), NTD (N-terminal domain), RBD, TM (transmembrane domain), SD1, and SD2 (subdomain 1 and 2), S1/S2 (S1/S2 protease cleavage site), S2′ (S2′ protease cleavage site), HR1, CH (central helix), HR2

**Fig. 2:**
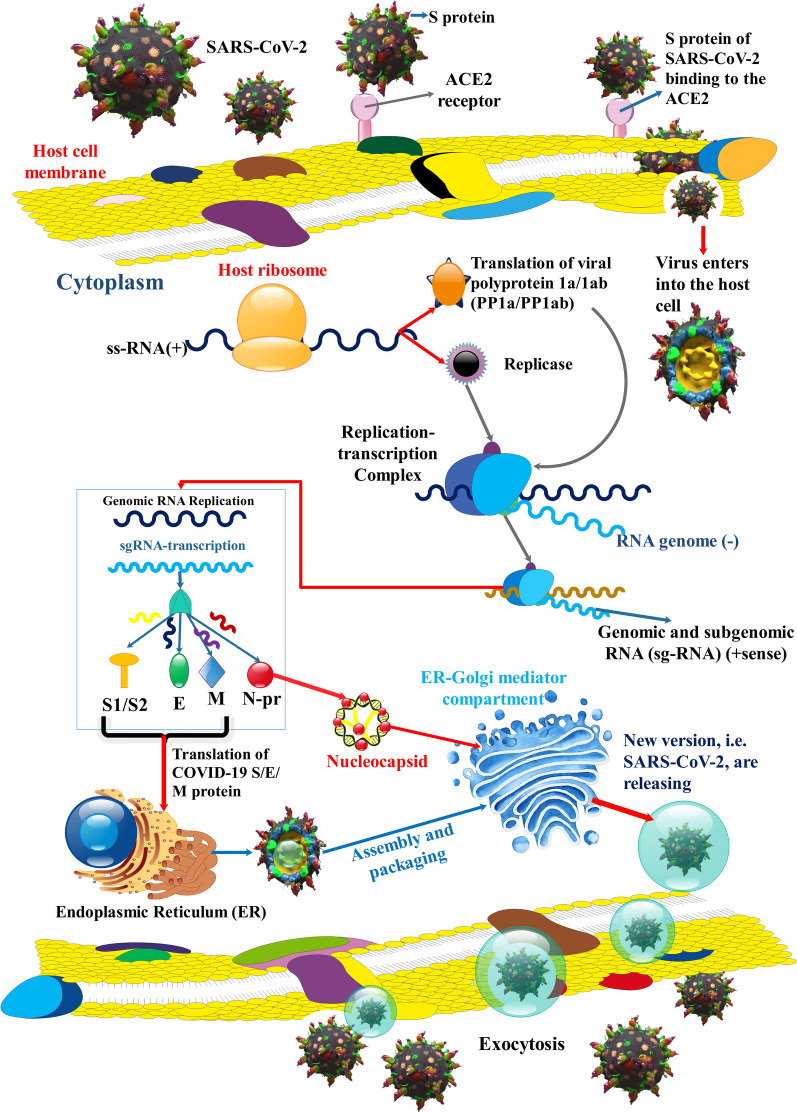
SARS-CoV-2 replication. SARS-CoV-2 S protein connects to ACE2 receptor to membrane fusion and discharges the viral genome into the host cell cytoplasm. After the entering the virus, the viral RNA is uncovered in the cytoplasm. ORF1a and ORF1ab are translated to produce pp1a and pp1ab that undergo further cleavage into smaller proteins containing RNA-dependent RNA polymerase, helicase, and nonstructural protein. RNA replicase–transcriptase complex (RTC) localizes to altered intracellular membranes isolated from the rough endoplasmic reticulum (ER) in the perinuclear area, where it generates (−) RNAs. Throughout replication, complete (−)RNA transcripts of the genome are generated and used as patterns for full (+) RNA genomes. Throughout transcription, a member of subgenomic RNAs (sg-RNA), containing those encoding S, M, E, and E protein, is generated by intermittent transcription. In this procedure, a nested set of (−)sg-RNA is generated that modify in length at the 3′ end and 5′-leader sequence, which is essential for translation. Then, this (−) sg-RNA are transcribed into (+) sg-mRNAs. Afterward, S, M, E, and N proteins are gathered in the nucleocapsid and viral envelope at the ER–Golgi intermediate compartment (ERGIC), then through exocytosis of SARS-CoV-2, released to vesicles [129–131]

Severe acute respiratory syndrome coronavirus 2 (SARS-CoV-2) led to the coronavirus disease 2019 (COVID-19), which was first reported in China in late 2019 [1]. The continued high prevalence of SARS-CoV-2 has prompted widespread worry worldwide [2, 3]. The population in the world with COVID-19 is on the rise, and it subsequently, led to a pandemic that infected more than 500 million people worldwide and killed more than 6.2 million by April 2022 [4]. One-third of the 3′ section contains SARS-CoV-2 genome-producing structural proteins, which include spike (S), envelope (E), membrane (M), and nucleocapsid proteins (N). Moreover, the viral genome, including 6 accessory proteins, is generated via ORF3a, ORF6, ORF7a, ORF7b, and ORF8 genes (3a, 6, 7a, 7b, 8, and 10 proteins) [5]. This virus uses S protein to bind to angiotensin-converting enzyme 2 (ACE2) in human cells. The ACE2 receptor is expressed in lung tissue and several extrapulmonary tissues, including the intestine, kidney, endothelium, and heart [6, 7]. A 3D-graphical illustration of the SARS-CoV-2 and structural proteins involved in the viral replication via membrane fusion or endocytosis on interaction S protein and RBD on ACE2 is depicted in Figs. 1 and 2.

SARS-CoV-2 in acute situations causes acute lung injury (ALI) and acute respiratory distress syndrome (ARDS) [8]. Common signs and symptoms of COVID-19 can include: fever, continuous cough, fatigue, chest and abdominal pain, shortness of breath, delirium, hoarse voice, skipped meals, diarrhea, and also decline and loss of taste and smell. However, in most infected people, it is asymptomatic [9]. In addition to the investigation of various antiviral drugs to combat COVID-19 (Table 1), based on the WHO reports, as of January 15, 2021, 64 vaccine candidates were under clinical evaluation to neutralize the SARS-CoV-2, and 173 candidate vaccines were in nonclinical studies. However, it might also be the case that these vaccines will be substituted at a future time by more novel candidates that display the same effectiveness but possess more tolerable reactogenicity patterns [10, 11]. Although efforts toward a vaccine as a foremost primary prevention strategy are progressing satisfactorily, early detection and timely treatment of critical cases are of vital significance [12–14]. COVID-19 targets the respiratory system mucosal cells and then transfects them to other kinds of cells, inducing a systemic cytokine, which leads to ARDS, disseminated intravascular coagulation (DIC), septic shock, and ultimately multiple organ damage [15–17]. Immune system problems have been diagnosed in treating infected people with severe COVID-19 pneumonia. The viral infection stimulates the immune system, causing a cytokine storm related to the severity of the disease. Besides, the severe form of the disease mostly involves people with chronic diseases or the elderly, who commonly have a small number of helper T cells and lymphocytes. Hence, to modulate the immune responses in these patients, a safe and efficient approach should be developed [18–20].

**Table 1:** The possible suppressors for SARS-CoV-2 infection

Drug/methods	Pathway	Previously used in	References
Camostat mesylate	TMPRSS2 suppressor, the effect on S proteins	Treatment of chronic pancreatitis, postoperative reflux esophagitis, and SARS-CoV	[5, 92]
Olumiant (Baricitinib)	JAK suppressor, the effect on S proteins	Treatment of rheumatoid arthritis	[93]
rhACE2 (APN01)	Decrease in proteolytic binding peptide angiotensin II	SARS-CoV infections, cardiovascular disease	[5, 94]
Umifenovir (Arbidol/ARB)	Preventing HA protein-interceded membrane fusion	influenza infection	[95]
T-705 (Favipiravir)	Inhibiting RdRp	influenza viruses, WNV, yellow fever virus, FMDV	[96, 97]
Abacavir (Ziagen)	RTIs (reverse-transcriptase inhibitors)	Treatment of HIV	[98]
Roflumilast (Daxas/Daliresp)	Preventing PDE4	COPD	[98]
Almitrine mesylate	is a respiratory stimulant	COPD	[98]
Chloroquine phosphate	IFN-α pathway	antimalarial	[99, 100]
Remdesivir	RdRp inhibitor	Treatment of Ebola and Marburg infections	[101]
Convalescent plasma	viral-specific antibodies	Ebola virus, H1N1, SARS-CoV-1, MERS-CoV	[102]

Stem cell therapy is an important therapy that has been used in different kinds of diseases. Stem cells (SCs) are primitive cells with self-renewal capability, and multidirectional differentiation capability, and can differentiate into a diversity of efficient cells or tissues. Presently, there are several uses and investigations for investigational stem cell therapy in severe patients of COVID-19, particularly mesenchymal stem cells (MSCs) therapy [21–23]. MSCs have immunomodulatory, anti-inflammatory, and regenerative attributes. In addition, MSCs can participate in the suppression of viral reproduction through various pathways. With the advance in regenerative and precision medicine, MSCs have been obtained from multitude tissues and utilized for particular tissue repair and renewal. Up to now, MSCs can be isolated from different kinds of adult tissues, mainly bone marrow, umbilical cord blood, adipose tissue, endometrium, uterine blood, embryos, etc. The safety and efficacy of MSCs therapy have been investigated in different clinical trials for the treatment of various diseases [24, 25]. The curative function of MSCs in SARS-CoV-2 patients seems to involve immunomodulation, improvement of repair/renewal, and suppression of viral reproduction via cell-to-cell interaction and paracrine acting. Since the prevalence of the SARS-CoV-2 infection, a collection of stem cell therapy clinical studies have been launched, and the outcomes demonstrated that MSCs not only result in a significant reduction of lung injury and time to improvement, however, as well as increase patient survival with good tolerance in the initial stage. The mixture of those properties makes MSCs attractive candidates for treating COVID-19 stimulated pulmonary inflammation. They might have a broader range of acts than medications, which commonly have a more restricted number of targets. The safety and effectiveness of MSCs have been reported in several clinical studies associated with the therapy of SARS-CoV-2 [26–28].

In this respect, due to their immunomodulatory and regenerative attributes, MSCs have been recently subjected to clinical studies to attain an acceptable and efficient remedial for COVID-19 [29, 30]. Therefore, in this review, we summarize the role and therapeutic potential of MSCs and the challenges ahead in using them to treat viral diseases with an emphasis on COVID-19.

## Immune responses in SARS-CoV-2

The receptor-binding domain (RBD) situated in S1 subunit connects to the ACE2 receptor in the zone of aminopeptidase N. In SARS-CoV-2, 6 RBD amino acids are essential for binding to ACE2 receptors, which causes detection of the host range of viral infection. These structural specifications of SARS-CoV-2 RBD enhance its ACE2-binding continuity [7, 31]. Moreover, despite the most effective viral replication, the COVID-19 virus did not remarkably activate types I, II, or III interferons (IFNs) in the lung tissue of COVID-19 patients [32].

**Fig. 3:**
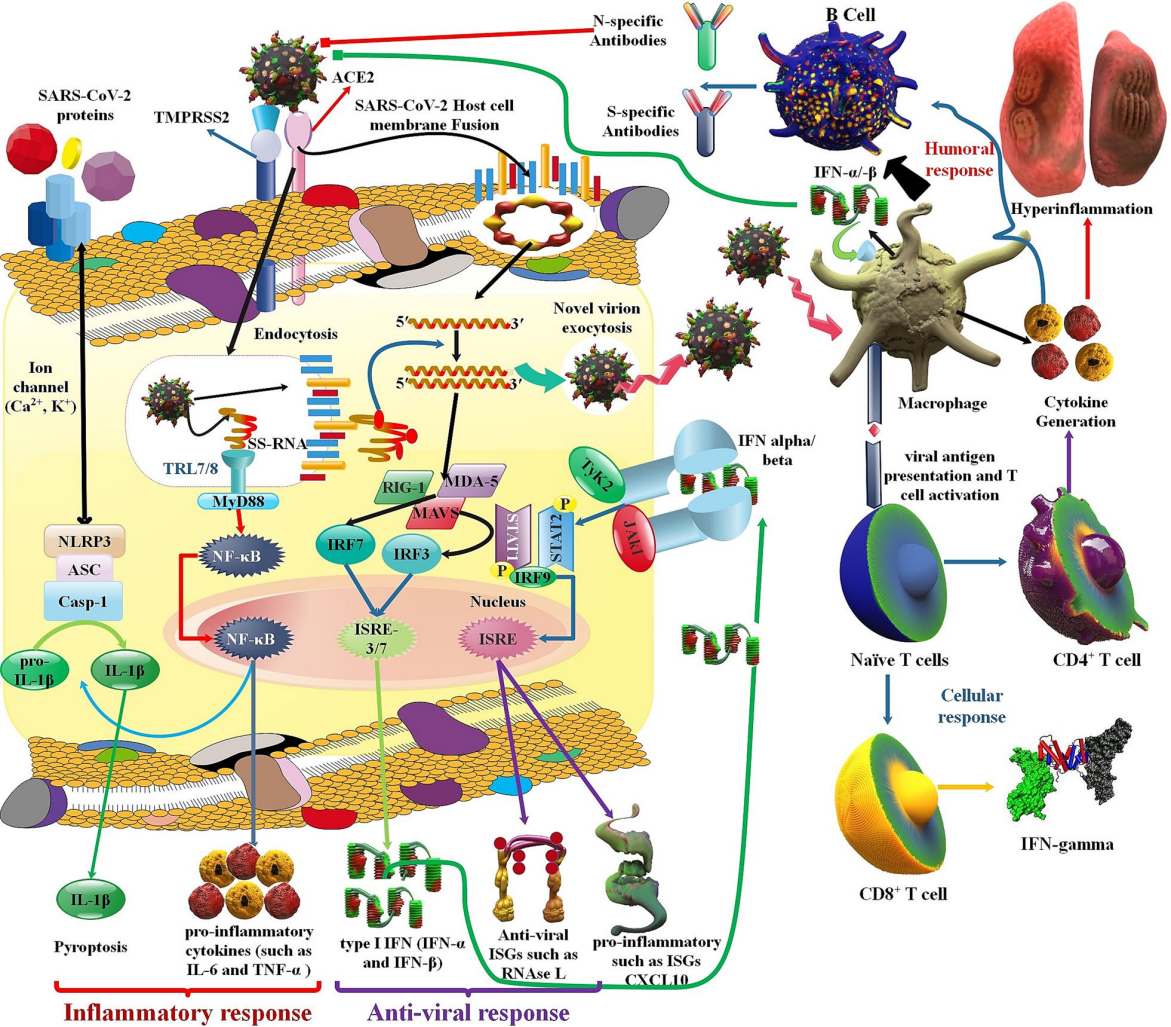
Possible immune system reactions in COVID-19 patients. To create an antiviral response, the innate immune system requires to diagnose the invasion of the viral, frequently via PAMPs. For the SARS-CoV-2, it is recognized that PAMPs in the shape of viral genomic RNA or the mediators in viral replication, such as dsRNA, are identified via either the endosomal RNA receptors, TLR3 and TLR7 and the cytosolic RNA sensor, and retinoic acid-inducible gene I/melanoma differentiation-associated gene 5 (RIG-I/MDA5). This detection occurrence triggers the downstream signaling cascade, including IRF3 and NF-κB, accompanied by their nuclear translocation. Type I IFN by interferon-α/β receptor (IFNAR), respectively, triggers the JAKs, STATs pathway, where JAK1 and tyrosine kinase 2 (TYK2) kinases phosphorylate STAT1 and STAT2. STAT1/2 organize a complex by IRF9, and simultaneously they transfer to the nucleus to initiate the transcription of IFN-stimulated genes (ISGs) with the regulation of IFN-stimulated response element (ISRE) comprising promoters [51]. Moreover, the SARS-CoV-2 is possible that trigger the inflammasome sensor, NACHT, LRR, and PYD domains-containing protein 3 (NALP3), leading to the release of the greatly inflammatory cytokine IL-1β and cause of pyroptosis, which is an inflammatory led to cell death. Envelope proteins and 3a-nsp of SARS caused the creation of the NLRP3 inflammasome [132, 133]

In the adaptive immune response, the body generates long-lasting plasma cells and memory T and B cells for rapid and exact detection of an antigen that the body has previously distinguished and commences a corresponding immune reaction [33]. The massive T cell replication is essential for prolonged immune reactions because the value of the primary clonal burst usually defines memory T cell numbers. In addition, vast cellular differentiation happens as these recently triggered T cells become strong antiviral effector T cells and, finally memory T cells. These T cells transfer to almost all tissues and inhibit viral infection via destroying infectious cells, generating cytokines, and recruiting other leukocytes by chemokine generation [34]. The period of memory CD8^+^ T cell growth happens in 2 steps after infection or vaccination [35, 36]. The prime stage starts when peripheral naïve CD8^+^ T cells activate in the exposure of antigen stimulation. Subsequently, these cells change into active cells and differentiate into effector cytotoxic T lymphocytes (CTLs) [37, 38]. As naïve CD8^+^ T cells differentiate into effector CTLs they can generate antiviral cytokines, including TNF-α, IFN-γ, and cytotoxic mediators, including granzymes (Gr) and perforin, and cytotoxic T cells quickly remove the viral infection [39]. Following antigen elimination, a secondary stage of T cell growth ensues. Most of the antigen-specific effector cytotoxic T cells perish through apoptosis, and the remaining effectors differentiate into memory CD8^+^ T cells [40]. CD4+ cell help (Th cell) is necessary for the produce of CD8^+^ CTL memory. The production and organizing of memory CD8^+^ T cells depend on “help” signals, which are provided via Th cells in the initial stage [41–43]. Furthermore, it helped memory CTLs express the effector program specification of helped initial CTLs upon recall with major histocompatibility complex (MHC) class I-limited antigens, presumably owing to epigenetic imprinting, and sustained mRNA expression of effector genes [44] (Fig. 3).

The efficient antiviral reactions of the host innate and adaptive immune system, such as the generation of different pro-inflammatory cytokines, the trigger of T cells, CD4^+^ and CD8^+^ T cells, are necessary for regulating the viral reproduction, inhibiting the extension of virus, inflammation and cleaning the infected cells. In addition, adaptive immune reactions are required for SARS-CoV-2 clearance; the, innate immune responses, such as macrophages, may contribute, in some cases, to the infection improvement [45]. Macrophages displayed a considerable generation of IL-6, which may contribute to the severe inflammation in SARS-CoV-2 infection [46–48]. The number of Th cells, including CD3^+^ T cells, CD4^+^ cells, and cytotoxic inhibitor T cells, including CD3^+^ and CD8^+^ cells, and regulatory T cells (T_regs_) are low compared to standard levels. In contrast, CD4^+^ cells and T_regs_ in acute patients are significantly lesser than in non-acute patients. Furthermore, the percentage of naïve helper T cells increases, while the rate of memory CD4+ and CD28+ cytotoxic inhibitor T cells decreased in acute COVID-19 [49]. And also, SARS-CoV-2 can cause non-acute or extremely acute respiratory syndrome with the consequent discharge of pro-inflammatory cytokines, such as IL-1β and IL-6. The connecting of SARS-CoV-2 to the toll-like receptor (TLR) leads to the release of pro-IL-1β, which is cleaved via caspase-1, after the inflammasome trigger and generation of active mature IL-1β. IL-1β is an intermediary between lung inflammation, fibrosis, and fever [50]. Furthermore, several innate cytokines, such as IP-10, MCP-1, CCL2, TNF-α, and MIP-1A, were elevated in COVID-19 patients, who required ICU care [51]. SARS-CoV-2-particular antibodies include IgM, IgG, and IgA (pan-immunoglobulin (pan-Ig)) antibodies against the N proteins, pan-immunoglobulin antibodies against the RBD, and IgG and IgA against the S1 subunit of the S proteins [52] (Fig. 3).

## Mesenchymal stem cells (MSCs)

Recently, bone marrow, adipose-isolated, synovium-obtained, and Wharton’s jelly-obtained MSCs are the most broadly utilized MSCs in tissue engineering. MSCs exist in approximately all tissues and are simply isolated. The multiple differentiation ability is one of the most essential specifications of MSCs. Moreover, various tissue origins influence the differentiation tendency and reproduction ability of MSCs [53–55]. In addition, because MSCs do not express significant histocompatibility compounds and immune-inducing molecules, they are not diagnosed via the immune system and do not result in graft rejection following transplantation. These attributes make them strong candidates in tissue engineering and regenerative medicine [56–59]. The Mesenchymal and Tissue Stem Cell Committee of the International Society for Cellular Therapy (ISCT) and Gene Therapy introduces MSCs as a large crowd with significant secretive, immunomodulatory, and homing attributes. The minimum criteria contain adherence to plastic, expressing particular surface markers, and ability of in vitro differentiation into adipocyte, chondrocyte, and osteoblast lineages [60, 61]. The immunomodulatory capability of MSCs involves T and B cell proliferation suppression, inhibition of cytokine generation, reduced NK cell function, and DCs maturation. MSCs with these specifications have been applied prosperously in different studies for inhibition of allogeneic graft rejection and the treatment of various diseases [62–64]. In addition, in tissue injury, MSCs can release paracrine and anti-inflammatory agents to repair tissue. MSCs induce metabolism not only via releasing a wide range of chemokines, growth factors, and cytokines, but as well as via the generation of several secretomes and proteomes [65, 66]. These agents have a significant act in immunomodulatory processes, mediating hematopoietic stem cell (HSC) engraftment and MSC differentiation, also controlling angiogenesis and apoptosis [67, 68].

### The immuno-related and other biological characteristics of MSCs derived from different tissues

Up to now, MSCs have been obtained from different origins. They have various proliferative potential, differentiation, and immune attributes. Researchers recommended that the proliferation amount of adipose-derived MSCs (AD-MSCs) was much more than that of bone marrow MSCs (BM-MSCs) or placenta-derived MSCs (P-MSCs) [69, 70], though P-MSCs could preserve a more prolonged proliferative stage and be persistently cultured for up to 160 days, with up to 64 population doublings. Reciprocally, BM-MSCs can only be persistently cultured for 60 days, with up to 12 population doublings. Another research showed that the population-doubling time in umbilical cord-MSCs (UC-MSCs) was much lesser than that of AD-MSSCs; however, UC-MSCs displayed earlier morphological alterations and a faster decrease in amplification capability [71, 72]. Human UC-MSCs can be attained without causing pain and show more rapid self-renewal. These MSCs have displayed significant anti-inflammatory, immunomodulatory and tissue repair acts with less immunogenicity, robust migratory capabilities, and neurotropic attributes, which makes them perfect candidates for allogeneic cell therapies to treat different diseases [73]. In another study, researchers showed that, although both BM-MSCs and AD-MSCs had immunomodulatory acts, differences in cytokine discharge resulted in ADSCs having a more powerful immunomodulatory action, equal to that of BM-MSCs. In addition, the immunomodulatory capability of P-MSCs was higher than BM-MSCs and AD-MSCs [74, 75]. MSCs obtained from infants and children's tissues can show even more significant biological attributes, including excellent proliferative capability, excellent differentiation ability, and longevity. In addition, children's MSCs have displayed strong immunomodulatory efficacy on the adaptive immune reaction and could even be utilized to inhibit viral infection [76].

Generally, MSC derived from different tissues shares the minimum defining specifications of cell surface markers, trilineage differentiation, and plastic adherence. But MSC differences based on tissue origin have been described for different species, more significant in association with their immunomodulatory features [77]. Several investigations have displayed that MSCs possess immunoregulatory attributes via modulating the reproduction and action of various immune cells, for instance, suppressing differentiation of monocytes into DCs, changing the cytokine profiles of DCs to lead to an upregulation of regulatory cytokines and downregulation of inflammatory cytokines. These immunomodulatory actions are mediated via cell–cell interactions and released cytokines, such as IFN-γ, indoleamine 2,3-dioxygenase (IDO), TGF-β, IL-6, IL-10, and prostaglandin E2 (PGE2). BM-MSC immunomodulatory properties were accepted by preventing T cell allogeneic proliferation stimulated in blended lymphocyte cultures or by non-particular mitogens [24, 78, 79]. BM-MSCs triggered via TNF-α and/or lipopolysaccharide (LPS) are able to discharging PGE2 into their microenvironment and stimulate macrophage generation of IL-10 in vitro and in vivo reprogramming macrophages into an anti-inflammatory profile. Other investigations have shown that macrophage polarization via human P-MSC, by glucocorticoid and progesterone receptor signaling. These results show that MSCs can induce M2-macrophages and monocytes via more than a single mechanism, and COX-2/PGE2 synthesis plays a role in the differential expression of anti-inflammatory molecules via myeloid cells [80].

## MSCs in viral infection

**Fig. 4:**
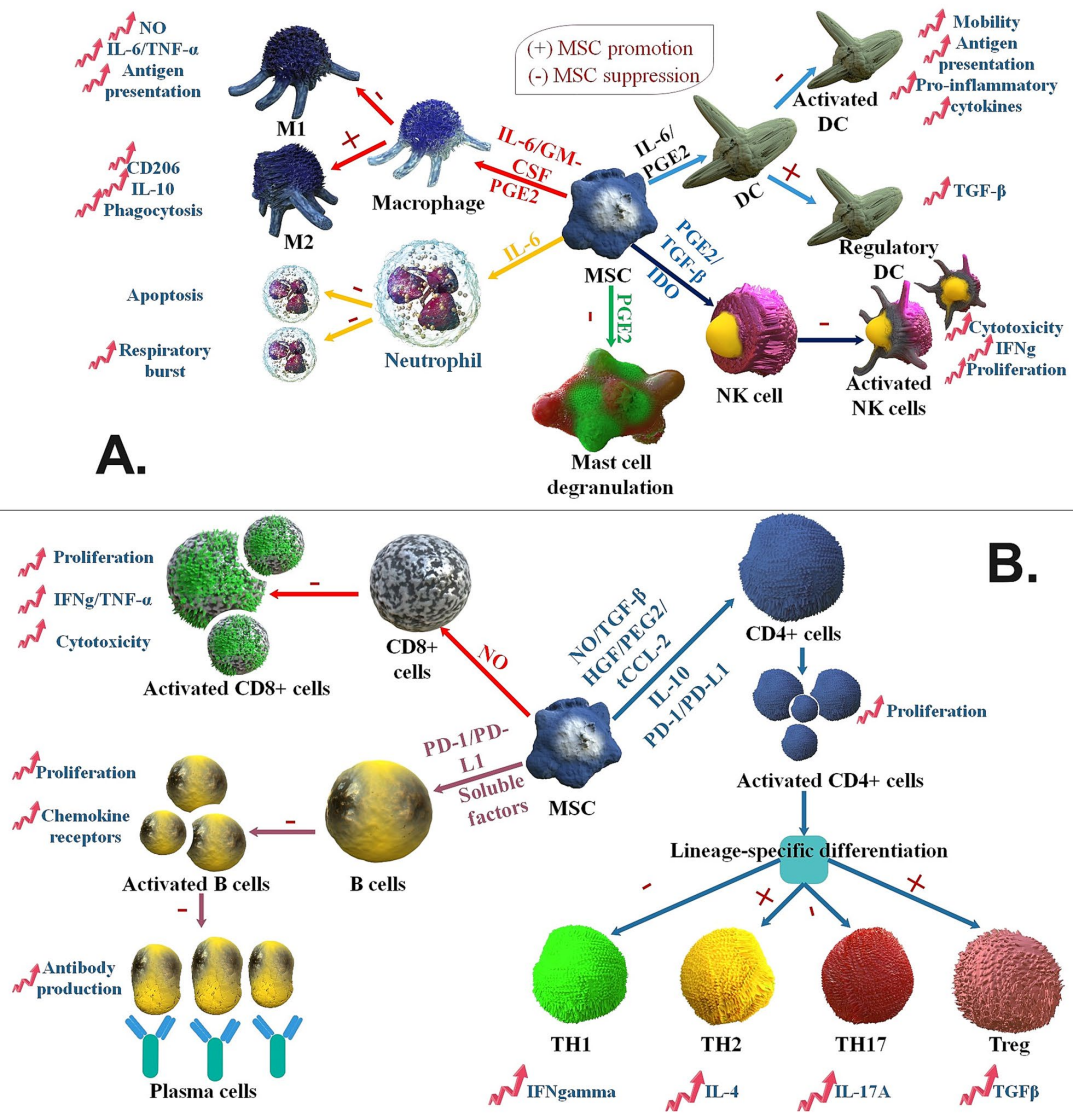
MSCs function as immunosuppression of the adaptive and innate immune system. **A** MSCs use various molecular pathways to inhibit innate immune cells. MSCs inhibit macrophage polarization to M1 via favors M2 polarization. MSCs suppress mast cell degranulation of histamine-comprising granules and suppress DC and NK cell activation, differentiation, and effector actions. MSC-isolated PGE2 chips in the whole of this efficacy. MSC-generated IL-6 inhibits neutrophil apoptosis and respiratory burst and helps suppress DC action. In the presence of GM-CSF and IL-6, MSCs also affect macrophage action, while IDO and TGF-β inhibit NK cell action. Moreover, MSCs favor the production of regulatory DCs. **B** MSCs suppress different facets of B cells acting, such as activation, reproduction, chemokine receptor expression, and differentiation to changing antibody-releasing plasma cells. Unknown soluble factors and programmed death-1 (PD-1)/PD ligand-1 (PD-L1) ligation intercede the efficacy of MSCs on B cells. MSC induced NO in reaction to inflammatory cytokine diagnosis to inhibit CD8+ T cell proliferation, cytokine generation, and cytotoxicity. In reaction to activation in a particular cytokine milieu, CD4+ T cells can differentiate into many effector crowds. MSCs generate soluble factors (IL-10, truncated CCL-2, PGE2, HGF, TGF-β, and NO) and membrane-bound molecules (PD-1 ligation) to inhibit T_h_ cell reproduction and the polarization of CD4^+^ T cells to TH17 and TH1 cells. MSCs favor the growth of anti-inflammatory T_reg_ and TH2 crowds [134]

Human MSCs are immunosuppressive and weakly immunogenic but may act as antigen-presenting cells (APCs) for CD4^+^ T cell reactions [81]. In viral infections, MSCs pulsed with peptides from viral antigens elicited IFN-γ and granzyme B discharge in specific CTLs and were lysed, though with low performance. In a viral infection, MSC intercedes immunoregulatory processes via suppressing the functions of various cell kinds. These MSCs activities include suppressing cytotoxicity versus viral-infectious cells, suppressing IL-2-driven NK cell IFN-gamma discharge and proliferation, decreasing pro-inflammatory cytokine discharge (IL-12, IFN-γ, TNF-α), enhancing IL-10 discharge, stimulating the development of T_reg_ cell, and downregulating the expression of the co-stimulatory molecule [82]. In addition, both cell–cell communication and discharge of soluble factors in MSC-interceded T cell inhibition are obvious overall in the literature, and several candidate intermediates have been shown: PGE2, IDO, NO, IL-27, TGF-, MCP-1/CCL2, HLA-G, and ICAM-1 [83] (Fig. 4).

Karlsson et al. demonstrated that EBV and CMV-induced proliferation and IFN-γ generation from peripheral blood mononuclear cells (PBMCs) were less influenced via third-party MSCs than the response to alloantigen and that MSCs had no efficacy on the development of EBV and CMV pentamer-particular T cells. Briefly, MSCs have less effective T cell reactions to EBV and CMV, which compares with their powerful immunosuppressive efficacy on alloreactive T cells. This information has the main suggestions for immunotherapy of graft-versus-host disease (GvHD) with MSCs and shows that the effective acts of virus-particular T cells may be retained following MSC injection [84]. In other studies, investigators demonstrated that cord blood-originated (CB) hematopoietic progenitor cells are positively differentiated into adult CD56+ CD94+ NKG2A+ NK cells on human CMV-infectious MSCs with significant greater antiviral cytokine generation compared to NK cells expanding on non-infected MSCs. In addition, the production of type 3 innate lymphoid cells (ILC3), defined via expression of the signature transcription agent retinoic acid receptor (RAR)-associated orphan receptor gamma (RORγt) and the generation of IL-22, was powerfully impaired via human CMV infection. These results are clinically related, given that ILC3 is dependent on preservation from GvHD after MSCs transplantation and HCMV reactivation, in turn, is dependent on increased incidence of GvHD [85]. Zhang et al. assessed the harmlessness and immunological reactions of human umbilical cord-MSCs (UC-MSCs) treatment in HIV-1-infected INRs. The UC-MSC transfusions favorably enhanced circulating naive and central memory CD4+ T cell levels and repaired HIV-1-particular IFN-γ and IL-2 generation in the INRs. These increases in immune repair were as well as related to the decrease in systemic immune triggers and inflammation in vivo. In addition, umbilical cord-MSC transfusions are well tolerated and can effectively increase host immune reconstitution in INRs, showing that such therapies may be utilized as a new immunotherapeutic method for reversing immune disorders in HIV-1-infectious INRs [86]. In other studies, investigators reported that exposure to MSCs or their conditioned media quickly enhanced HIV-1 viral protein called p24 generation from the latently-infected U1 (MAC) & ACH2 (THL) cells. In addition, exposure to MSCs enhanced HIV-1 long terminal repeat (LTR) directed gene expression in the MAC and THL reporter cell lines, J-Lat and U937-VRX (9.2), respectively. The MSC-interceded latency-reactivation was related to the NFκB and PI3K pathways. Consequently, the pre-clinically tested suppressors of NFκB (CDDO-Me) and PI3K (PX-866) inhibited MSC-interceded HIV-1 reactivation [87]. The SDF-1 (also known as CXCL12)/CXCR4 pathway is essential for regulating MSC replication, immigration, and differentiation. Moreover, HIV envelope glycoprotein gp120 suppresses CXCL12-stimulated chemotaxis via decreased expression and function of CXCR4 in monocytes, B, and T cells. However, Gp120 increased MSC CXCR4 expression, which increased the effects of CXCL12 in stimulating chemotaxis extracellular signal-regulated kinases (ERKs), and focal adhesion kinase (FAK)/Paxillin pathways were extreme-triggered, thereby simplifying actin stress fiber reformation. As a result, gp120 from both T cells and monocytes-tropic HIV strains upregulated CXCR4 expression in MSCs, leading to increased MSC chemotaxis in reaction to SDF-1 [88]. Parvovirus B19 (B19V) has been recognized as stimulating transient erythroid aplasia, cytopenia, and aplastic anemia. This virus persists in human BM-MSCs of certain immunocompetent persons many years afterward initial infection. In B19V-infectious erythroid progenitor cells, the virus stimulates the transactivation of IL-6 and TNF-α gene expression. In a study, results of the analysis showed that considerable enhancement of TNF-α and IL-6 gene expression, respectively, and reduce in burst forming unit-erythrocyte and colony-forming unit-erythrocyte numeration. As a result, stimulating inflammatory cytokines gene expression in B19V-infectious BM-MSCs might affect BM microenvironment and hematopoiesis [89]. In a recent study, researchers examined the lack of side effects and effects of the composition therapy of plasma exchange and UC-MSCs transplantation for treatment of the HBV-dependent acute-on-chronic liver failure. In this treatment method, the whole bilirubin, aspartate aminotransferase, alanine aminotransferase, and model for end-stage liver disease (MELD) score were remarkably reduced. As a result, UC-MSCs, together with PE treatment, had well harmlessness; however, they cannot importantly enhance the short-time prognoses of HBV-ACLF patients compared with the one therapy [90]. MSCs mediate their immunoregulatory and tissue restoration actions via discharging paracrine agents, such as extracellular vesicles (EV). MSC-EVs obtained from an MSC-conditioned medium (CM) via ultracentrifugation. MSC-EVs suppressed the hemagglutination action of swine, human, and avian influenza virus at concentrations of 1.25–5 μg/ml. MSC-EVs suppressed influenza virus reproduction and virus-stimulated apoptosis in lung epithelial cells [91]. In addition, EVs-MSC delivers a diversity of molecules, including mRNA, microRNA, and even organelles, including mitochondria to improving ARDS in preclinical models [92] (Table 2).

**Table 2:** MSCs in viral infection

Viral infection	Virus effects on MSCs function in viral infection	References
EBV and CMV	These viruses-induced proliferation and IFN-γ generation from peripheral blood mononuclear cells (PBMCs) were less influenced via third-party MSCs than the response to alloantigen and that MSCs had no efficacy on the development of EBV and CMV pentamer-particular T cells. MSCs have less effective T cell reactions to EBV and CMV, which compares with their powerful immunosuppressive efficacy on alloreactive T cells	[40]
Human CMV	CB hematopoietic progenitor cells are positively differentiated into adult CD56+ CD94+ NKG2A+ NK cells on human CMV-infectious MSCs with significant greater antiviral cytokine generation compared to NK cells expanding on non-infected MSCs	[41]
HIV-1	The systemic inflammatory indexes, including high amounts of pro-inflammatory cytokines, chemokines, and growth factors, were reduced via UC-MSC treatment in these immune non-responders cases	[42]
HIV-1	Exposed to MSCs or their MSC-CM quickly enhanced HIV-1 viral protein called p24 generation. Besides, MSCs enhanced HIV-1 LTR, targeted gene expression. MSC-interceded latency-reactivation cycle was related to the PI3K and NFκB pathways. Consequently, the pre-clinically experimented suppressors of PI3K (PX-866) and NFκB (CDDO-Me) repressed MSC-interceded HIV-1 reactivation	[43]
HIV	HIV envelope glycoprotein Gp120 suppresses CXCL12 activated chemotaxis via decreased the expression and action of CXCR4 in monocytes, B, and T cells. Gp120 led to increasing MSC CXCR4 expression. As a result, it is possible that CCR5-knockout is helpful in HIV infection treatment	[44]
Parvovirus B19	This virus persists in BM-MSCs of some immunocompetent persons many years afterward the first viral infection. Also, it led to enhancing the expression amounts of interleukin-6 and TNF-alpha severally and reduced BFU-E and CFU-E numeration	[76]
HBV	Plasmapheresis and UC-MSCs transplantation are used in the therapy of HBV-dependent ACLF. The results of this treatment method were negative and unsuccessful	[47]
Influenza virus	MSC-EVs suppressed the hemagglutination function of swine, human, and avian influenza viral about condensations of 1.25–5 μg/ml and repressed viral-caused apoptosis in lung epithelial cells of a pig	[49]

## MSCs as a therapeutic option for COVID-19

One of the benefits of using MSCs in the treatment of COVID-19 is that ACE2 and TMPRSS2 are not expressed in human MSCs obtained from both adult and fetal tissues, so they are not infected by the SARS-CoV-2. Moreover, use of MSCs therapy in lung diseases leads to better improvement of disease-related parameters in ARDS. Besides, MSCs lead to the preservation of alveolar epithelial cells in ALI and ARDS. MSC contributed to SARS-CoV-2 therapy, including immunomodulatory effects and differentiation capability [93–95]. Following intravenous (IV) injection, a considerable crowd of cells accumulates in the lung, which besides from immunomodulatory efficacy, could preserve alveolar epithelial cells, regenerate the pulmonary microenvironment, inhibit pulmonary fibrosis, and treat functional lung disorders. In addition, in SARS-CoV-2 infection, these MSCs can improve the lungs' microenvironment. Furthermore, MSCs are derived from several mature tissues, including BM, peripheral blood and adipose tissues, and neonatal birth-related tissues, such as cord blood (CB), amniotic fluid (AF), Wharton jelly (WJ), umbilical cord (UC), and placenta, and then collected for feasible future usage [96]. Following the MSCs therapy in SARS-CoV-2 infection, the CRP was reduced, the number of peripheral lymphocytes enhanced, and overactivated cytokine-secreting immune cells disappeared (including CXCR3^+^ NK cells, CXCR3^+^CD8^+^ T cells, and CXCR3^+^CD4^+^ T cells). Furthermore, a group of CD14+ CD11c+ CD11bmid regulative-DC crowd enhanced after MSC therapy, and a level of TNF-α was reduced with the simultaneous increase in the level of the IL-10 [97]. In COVID-19, despite a large number of infected mature and aged people, fewer kids and teenagers are infected. The higher existence of MSCs produces a rapid reaction to suppress the generation of pro-inflammatory cytokines and interleukins, including TNF-α, IFN-y, IL-6, IL-2, and IL-1. The anti-inflammatory processes of MSCs occur in 2 supplementary stages, first affecting the monocyte differentiation to DCs and M2, and second, by stimulating tolerant phenotypes of naive and effector T cells and suppressing antibody generation via B cells together with an inhibiting acting on NK cell reproduction and NK cell-interceded cytotoxicity [98].

In addition, MSCs discharge several essential agents, including hormones and cytokines for tissue repair, known as secretomes. IV administration of secretome is spread to the lungs and stays constant in the body. Furthermore, utilizing secretome has its benefits contrasted to using MSCs themselves, particularly in SARS-CoV-2 infection. The MSC-obtained secretome has benefits in storing situations, including constancy, and it is cheaper than MSCs. In addition, this secretome can trigger the endogenous SCs and progenitor cells, control the inflammatory reaction, induce angiogenesis and repairing of the extracellular environment, inhibit apoptosis, intercede chemoattraction, and decrease fibrosis. The MSC secretome also prevents the danger of teratoma generation [99].

### Therapeutic characteristics of MSCs derived from different tissues in SARS-CoV-2 infection

MSCs can control inflammation via a chain of procedures such as increasing the absorbency of T_regs_, such as CD8^+^CD28^−^ T lymphocytes and CD4^+^CD25^+^ FoxP3^+^ T lymphocytes, suppressing excessively high reproduction and differentiation of B cells, the maturity of DC, and improving macrophage anti-inflammatory phenotypic polarization. Consequently, the modulation regulatory role of MSCs in the immune system (immunomodulation) could be crucial in decreasing the incidence of cytokine storm syndrome in COVID-19 infection. RADWAN SM et al. demonstrated that AD-MSC can suppress the NF-κB signal pathway, decrease the expression of pulmonary pro-inflammatory agents, reduce pulmonary inflammation, and eventually inverse the procedure of amiodarone-influenced pulmonary fibrosis in rats. The CM generated via Ad-MSC cultivation in *xeno-free* cultures could be an efficient remedial alternative for patients with weak clinical improvement or those in need of a severe treatment strategy. Moreover, van Asten’s et al. showed that the FGF derived from the Ad-MSC secretome displayed viral replication suppression characteristics. As a result, this type of MSCs may be used to treat the SARS-CoV-2 [100, 101]. Researchers proved that the use of the adoptive transfer of allogeneic human UC-MSCs in 65-year-old women with acute COVID-19 leads to improvement in their infection. In this method, this type of MSCs generated following a good manufacturing practice (GMP) situation was implemented through IV injection 3 times (5 × 10^7^ cells each time). Also, antibiotics were given to inhibit infection, and thymosin α1 was also given. Following the first injection, no evident adverse events were detected, showing it was well tolerated. Afterward, during the secondary injection, the levels of serum bilirubin, CRP, alanine aminotransferase (ALT)/aspartate aminotransferase (AST) were slowly decreased, along with some other essential signs as well as improved. Significantly, CTLs, Th cells, and CD3+ T cells were also considerably enhanced to ordinary levels [102]. The researcher has shown that the useful function of MSCs in SARS-CoV-2 could be ascribed to their capability to discharge bioactive lipids (BALs), including lipoxin A4 (LXA4), prostaglandin E2 (PGE2), and other analogous BALs. Additionally, PGE2, LXA4, and their precursor's AA (arachidonic acid), gamma-linolenic acid (GLA), and dihomo-gamma-linolenic acid (DGLA) suppress the generation of TNF-α and IL-6, the immune checkpoint inhibitory (ICI) treatment, sepsis, and ARDS. Consequently, injections of applicable rates of GLA, DGLA, AA, PGE2, and LXA4 are of significant therapeutic advantage in SARS-CoV-2 infection, ICI treatment, and other inflammatory situations, containing but not confined to sepsis [103]. Investigators used hUC-Wharton’s jelly-obtained MSC (WJCs) in the treatment of acute COVID-19 patients. As a result, IV injection of WJCs-MSCs in the patient increases CD3+, Th cell, and cytotoxic T cell levels, and the amount of CRP, TNF-α, and IL-6 is significantly reduced following hWJC-MSCs therapy [104] (Table 3).

**Table 3:** Several kinds of MSCs for COVID-19

Stem cell	Function in COVID-19	References
UC-MSCs	Implemented IV infusion to 3 times (5 × 10^7^ cells each time) caused recovery 65 aged women with acute SARS-CoV-2 infection	[55]
MSC-EVs	MSC-Evs have different and broad types of origins. Also, they contain molecules of an election, including a siRNA, microRNA, or protein which led to improvements in different tissue injuries	[56, 57]
BM-MSC-Exos	Only one 15 mL IV injection dose of Exos (ExoFlo™) in acute condition of COVID-19 patient. As a result of this treatment, 83% of cases improved	[58]
MSCs	In MSCs treatment, the CRP reduced, the number of peripheral lymphocytes enhanced, and overactivated cytokine-secreting immune cells disappeared (including CXCR3^+^CD4^+^ T cells, CXCR3^+^CD8^+^ T cells, and CXCR3^+^ natural killer cells). Also, a group of CD14^+^CD11c^+^CD11bmid regulative-DC crowd was enhanced, and the amount of TNF-α was reduced by a simultaneous increase in the level of the CSIF	[59]
SCs/MSC-like cells (SP and GLI1 cells)	Inhabitant SCs/MSC-similar cells (SP and GLI1 cells) have a significant function in pulmonary fibrosis. Their quantities decrease in mouse and SARS-CoV-2 infected persons by this complexity contrasted with average persons. And also, MSCs can influence immunosuppression and tissue repair	[17]
MSCs-dependent on hACE21-740-Fc	MSCs-dependent on scFv-IL6R-Fc and hACE21-740-Fc transfer method, which prepared a possible fast election to immediate clinic remedial requirement of SARS-CoV-2 infected cases	[60]
MSCs	MSCs may control increasing the absorbency of T_regs_, including CD4^+^CD25^+^FoxP3^+^ T lymphocytes and CD8^+^CD28^−^ T lymphocytes, suppressing excessively high reproduction and differentiation of B cell, the maturity of DC, and improving macrophages to anti-inflammatory phenotypic polarization	[61, 62]
AD-MSC	AD-MSC can repress the NF-κB signal pathway, decrease the expression of pulmonary pro-inflammatory agents, diminish pulmonary inflammation, and eventually inverse the procedure of amiodarone-influenced pulmonary fibrosis in rats. Moreover, the FGF, derived from the Ad-MSC secretome, displayed virus enhancement suppression characteristics	[61, 62]
MSCs	PGE2, LXA4, and their precursors, AA, DGLA, and GLA inhibit the generation of IL-6 and TNF-α, and also in the ICI treatment method, sepsis and ARDS are helpful in the treatment for SARS-CoV-2 infection. Moreover, GLA, DGLA, and AA disable enveloped viral such as SARS-CoV-2	[63]
WJCs-MSCs	IV injection of WJCs-MSCs in the patient, the levels of the CD3+, helper, and cytotoxic T cell are increased. Also, the measure of C-reactive protein, TNF-alpha, and interleukin-6 is remarkably reduced	[64]

### The potential usage of exosomes derived from MSCs (MSCs-Exo) and MSCs-EVs in SARS-CoV-2 treatment

Exosomes (Exos) are released from all cells, including SCs. Exos have displayed molecular likeness with their mother cells; therefore, it has been hypothesized that MSCs-Exo can be injected into the injured region for neovascularization and tissue regeneration. Exos are membrane-surrounded EVs with a diameter ranging from 30 to 100 nm. Investigations of Exos have shown that they are constant at low storage temperatures for long-term, and this survival level and constancy become them better than the parent cell. Moreover, when contrasted to cell therapy, Exos and EVs provide an attractive, safe, and hopeful therapeutic method because they have no nuclei, which saves them from the danger of tumor creation and each type of mutation. Despite not having a nucleus, Exos have all the necessary growth factors and biosignals that helps in repair of injured tissues. Evaluation of Exos’ genomics and proteomics information showed that the quality of their messenger RNA, microRNA, transfer RNAs, and protein is first rate [105]. MSC-Exos can suppress T helper cells, CD8^+^ T cells, and NK cells. MSC-Exos suppressed T cells expressing IL-17 and stimulated IL-10 expressing regulatory cells involved in the inhibition of infection. MSC-Exos as well as helps in inhibiting the differentiation of T_h_ cells and CTLs via discharging molecules such as TGFβ and inhibit inflammation in vivo. MSC-Exos could change macrophages from the M1 to the M2 phenotype, further inhibiting pro-inflammatory states. Moreover, sepsis is a severe fatal agent in SARS-CoV-2 infection, and therapy by MSC-Exos has enhanced the amount of improvement in the sepsis mice model. Simultaneously, MSC-Exos as well as inhibited the discharge of the pro-inflammatory agents, including TNF-α, IFN-γ, IL-6, IL-17, and IL-1β, and increased the release of anti-inflammatory agents, including IL-4, IL-10, and TGF-β. In addition, MSC-Exos decreased the number of chemokines in the serum when administered. The immunomodulatory efficacy of MSC-Exos has also been ascribed to their anti-inflammatory load, including IDO, HLA-G, PD-L1, and galectin-1 [106]. In another study, investigators used a single 15 mL IV dosage of allogeneic BM-MSC-derived Exos (ExoFlo) to treat acute SARS-CoV-2 infection. As a result, 71% of the infected cases improved, 13% stayed severely sick although constant, and 16% died for reasons not associated with the MSCs therapy. In addition, laboratory amounts revealed significant recovery in absolute neutrophil number and lymphopenia with mean CD3^+^, CD4^+^, and CD8^+^ cell numbers enhancing [107]. New studies are hopeful and encouraging for more investigations and clinical usages of AD-MSCs and MSCs-Exos for the therapy of SARS-CoV-2 infection. Certain clinical studies were recorded and facilitated the path to their parent cells protected via the harmlessness and effectiveness of their provision. Furthermore, increasing promising results using these extracellular vesicles to treat many diseases have been reported (Table 4) [108].

**Table 4:** List of recorded MSCs-Exos-based clinical studies for inhibiting COVID-19

Study Description	Study design	Primary Outcome Measures	Recruitment Status	Study identifier
This pilot clinical study will discover the harmlessness and performance of aerosol nasal route administration of the exosomes isolated from allogenic AD-MSCs (MSCs-exosome) in acute COVID-19 patient	Clinical Trial. N/A, 30 participants 5 times aerosol nasal route administration of MSCs-Exos (2.0 × 10^8^ nanovesicles/3 ml from 1–5 day) 18–75 Years Phase I	1. Safety assessment within 28 days next to the primary therapy, containing the frequency of adverse effects and acute negative reaction 2. Performance assessment in 28 days, containing the time to clinical recovery	Completed	NCT04276987
Therapy of SARS-CoV-2 Patient, who are at primary phases of pulmonary infection by SARS-CoV-2-particular T cell-isolated Exos (CSTC-Exos) to control infection development	Clinical study. N/A, 60 patients. Nasal route administration of CSTC-Exo therapy will be used five times each day (2.0 × 10^8^ nanovesicle / 3 mL; on day 1 to day 5) 18–75 Years Phase I	1. Harmlessness evaluation: Adverse effect and acute adverse reactions in 28 days 2. Effectiveness evaluation 28 Days 3. The amount of improvement Ventilator-free, in 28 days	Active, not recruiting	NCT04389385
To assess the harmlessness and effectiveness of intravenous injection of BM-ExoFlo, against placebo as therapy for regulating ARDS in severe SARS-CoV-2 infected persons	A multicenter, placebo-controlled, randomized clinical study. 120 members Experimental Dosage 1: Normal saline solution 90 mL and ExoFlo 10 mL, 800 × 10^9^ nanovesicle Practical dosage 2: normal saline solution 85 mL and ExoFlo 15 mL, 1.2 × 10^12^ Vesicles 18 Years to 85 Years Phase II	1. All-cause death 2. Median days to improvement	Completed	NCT04493242
This trial has been created based on the literature, data about the ongoing tests NCT04276987 (A Pilot Clinical trial on Inhalation of MSC-Exos improving acute SARS-CoV-2 infection), and NCT04384445 (Organicell flow for infected persons by SARS-CoV-2), Patent No 271036826 of 2019. A technique for obtaining miRNA-containing exosomal multipotent MSCs for application in cosmetic and pharmaceutical productions	Interventional (Clinical Trial), 30 members, Randomized, 3 groups. All qualified trial members are randomized, double-blinded, to either the 2 therapy or placebo group Inject twice a day for up to 10 days, aspiration of 3 ml specific solution included 0.5–2 × 10^10^ nanoparticles (Exos) 18 Years to 65 Years Phase I Phase II	1. Safety evaluation, including side effects, will be recorded. Side effects will be checked during all trials 2. Safety evaluations, including side effects during the inhalation routs, will be recorded	Completed	NCT04491240

**Fig. 5:**
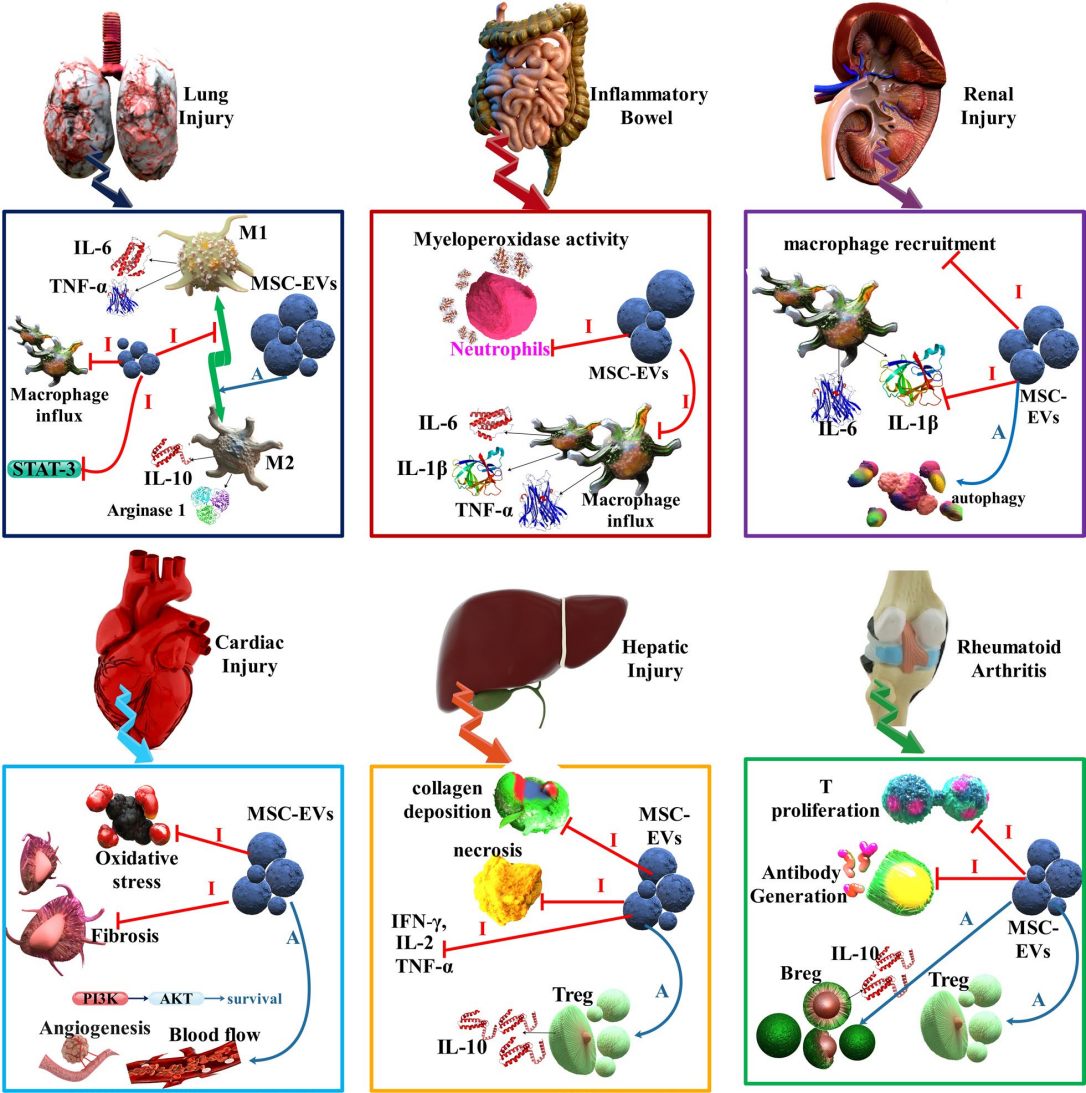
Different functions [Inhibition (I: red arrows) and Activation (A: blue arrows)] of MSC-EVs in various tissues injury. BM-MSCs Exos reduced macrophage influx, repressed inflammatory intermediaries, including MCP-1, and suppressed STAT3 signaling in the lung injury. Besides, UC-MSC EVs recovered lung functions by bronchopulmonary dysplasia through increasing M1-M2 change in macrophages showed by decreased IL-6, TNF-α, CCL5, and enhanced arginase 1. BM-MSC EVs diminished bowel injury, limited myeloperoxidase action in the colon, reduced IL-1β, TNF-α, and inducible nitric oxide synthase (iNOS) amounts in the bowel. Moreover, upon UC-MSC EVs therapy, macrophage penetration to the tissue was decreased along with downregulation of TNF-α, IL-1β, IL-6, IL-7, and iNOS in colon tissue and spleen. BM-MSC EVs are protected in a murine renal I/R injury model and the preservative efficacy was associated with the C–C chemokine receptor type 2 transported on the exosome surface, which could separate the chemokine (C–C motif) ligand 2 (CCL2) and thus damaged macrophage recruitment and activation. Moreover, UC-MSC EVs decreased the amounts of IL-1β and TNFα while increasing autophagy. Human embryonic stem cell-isolated MSCs decreased oxidative stress, enhanced myocardial livability via the triggering of the PI3K/Akt pathway, and therefore increased cardiac activity. Besides, BM-MSC EVs enhanced angiogenesis and increased blood circulation in myocardial infarction models while preventing T cell proliferation. HUC-MSCs EVs were displayed to decrease hepatic inflammation while reducing TGF-β amounts and collagen deposition in murine liver fibrosis. Moreover, BM-MSC EVs infusion decreases IFN-γ, IL-1, IL-2, TNF-α expression, enhances T_reg_ level, and reduces necrosis in the liver. MSC-EVs in rheumatoid arthritis suppress T cell proliferation and also promotion of T_reg_ cells, B_reg_ cells [110]

**Fig. 6:**
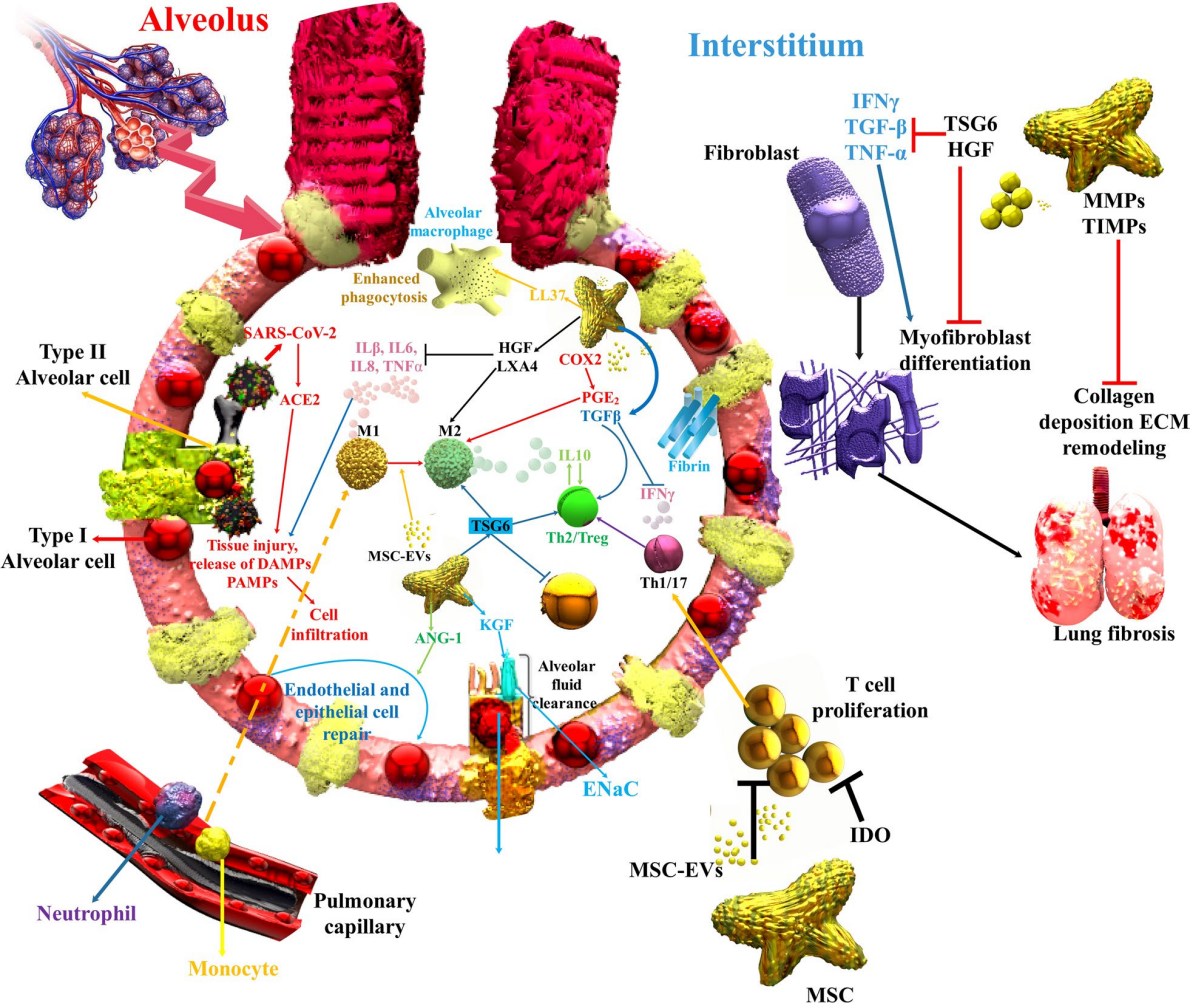
COVID-19 leads to cell and damage injury, discharge of danger-associated molecular patterns (DAMPs), PAMPs, and inflammatory intermediates that increase immune cell penetration. Injected MSCs-EVs can decrease inflammation and induce tissue regeneration. Significantly, the good efficacy of MSCs is displayed to be related to the decrease in TNF-α, interleukin-1/6 via the discharge of HGF, PGE2, lipoxin A4 (LXA4), and TSG-6, inhibition of inflammatory T cell proliferation via indoleamine 2,3-dioxygenase expression, switch from Th1 and Th17 reactions to Th2, and suppression of monocytes and myeloid DCs maturation. MSCs stimulate M2 polarization via juxtacrine signaling and paracrine agents, including HGF, PGE2, and TSG-6, leading to a monocyte helping enhance anti-inflammatory IL-10, which synergistically induces T_reg_ cells and activates tissue regeneration pathways. Additionally, MSC involvement in several mechanisms that increase lung fibrosis leads to protecting efficacy, as shown in various lung damage models. Notably, MSCs decrease the damage-associated alveolar edema and endothelial permeance via the discharge of keratinocyte KGF to increase the sodium-affiliate alveolar fluid clearance via epithelial sodium channel (ENaC) and have anti-apoptotic and anti-oxidative pathways to repair cytokine-injured alveolar type II cells and epithelial and endothelial damage via the discharge of angiopoietin-1, lipoxin A4 (LXA4) and TSG-6 [135]

In addition, using MSC-EVs is a helpful treatment method for treating the COVID-19. MSC-EVs have a different and wide range of origins, such as BM, periodontal ligament, placenta, amniotic fluid, UC, peripheral blood, AD tissue, and gingival tissues. Furthermore, these extracellular vesicles could be pay-loaded with a molecule of select, such as siRNA, microRNA, a related to what is considered most dependent on interrupting the processes of the virus in the cell and thus preserving the airways and lungs [109]. Moreover, MSC-EVs have various functions in different tissue injuries, including cardiac, renal, hepatic, lung, and inflammatory bowel in the SARS-CoV-2 [110] (Figs. 5 and 6). Engineered MSCs slowly release their compounds via exocytosis or within EVs in the microenvironment of injured tissues. The use of loaded MSCs reduces the systemic collateral toxicity of medications, prepares a continued discharge system that enhances the time of medication efficacy in the tissue, and acts in a targeted method. Direct cell–cell connection and CM are two methods for administering medication-loaded MSCs. In addition, MSCs can discharge encapsulated medications via employing EVs that carry medicines among MSCs and host cells. MSCs were injected effectively for lung transfer of IFN-β and IFN-α, which are the best candidates for COVID-19 therapy in various stages of clinical studies. VEGF have a significant function in brain inflammation in SARS-CoV-2 infection. Designed MSCs can discharge solvable Fms-associated receptor tyrosine kinase 1 (Flt-1), VEGF receptor that competitively suppresses the agent from connecting to the organ receptors [111]. In another study, investigators researched whether the MSC-EVs microRNA load can regulate the intensified cytokines, cell dying and clotting disorders existing in acute SARS-CoV-2 infection. Via bioinformatics methods, 4 data collections of microRNA, utilizing several SC tissue origins (BM, UB, and AD tissue), and one data collection of mRNA (BM) were studied. The result was a forecast of 258 microRNAs for intensified cytokines and chemokines, 266 microRNAs for cell death genes, and 148 microRNAs for clotting cascades. Specific microRNAs may reduce inflammatory factors while also preventing cell death genes and essential agents of the clotting cascade, consequently inhibiting tissue injury and clotting disorders. So, the MSC-EVs, because of their heterogeneous load, are a possible multi-target method capable of enhancing the survival of acute COVID-19 patients. In addition, several target features of the MSC-EVs microRNA load could benefit, in contrast to treatments targeting just one target, including Tocilizumab or Anakinra (anti-IL1R). These treatments are used to reduce inflammatory markers, owing to their interplay with several mechanisms. They improve the infected person's results by reducing tissue injury and decreasing coagulation triggering and thrombi formation [112–114] (Fig. 6).

## MSCs' clinical research progress in SARS-CoV-2 infection

MSCs have been seriously studied in clinical studies for many situations. There are more than 3987 completed, ongoing, and planned trials associated with MSCs (Clinicaltrials.gov, 2022) [115]. Currently, there are no efficient therapies accessible for acute COVID-19 patients. However, MSCs transplantation is an emergency therapy technique that could be used to address this issue and is newly being evaluated in clinical studies. As of April 18, 2022, 394 MSCs studies are recently being verified in clinical studies to treat SARS-CoV-2 [116] (Table 5).

**Table 5:** List of recorded MSCs treatment-based clinical studies for COVID-19 (https://clinicaltrials.gov/)

MSCs types	Study design	Population	Results or aims	NCT Number
WJ-MSCs	Phase I Interventional	5 (18 years and older)	The first result shows that recovery of clinical signs	NCT04313322
Autologous AD-MSCs (Celltex-Ad-MSCs)	Phase II multicenter, double-blind, randomized, placebo-control	200 (18 years and older)	The first result shows that this method have no side effects and tolerability	NCT04444271
MSCs	Prospective, Randomized Phase II	20 Age: 18–65	Occurrence of SARS-CoV-2 in trial and control group first result: total survivorship at 30 days after-treatment	NCT04336254
Allogeneic Human Dental Pulp Mesenchymal	Single-center, prospective, randomized	20 Age: 18 − 65	The first result shows that time for clinical recovery	NCT04429763
UC-MSCs	Phase II	30 Age: 18 − 79	The first result shows that clinical decline or dying	NCT04416139
Allogeneic UC-MSCs	Pilot experiment	10 (18 years and older)	The first result: clinical, biochemical, inflammatory, and immune alters	NCT04315987
Allogeneic HB-AD-MSCs	Phase 2 (clinical trials performed early in Phase I)	90 (18 years and older)	Early Consequence: alteration in the clinical situation	NCT04348435
MSCs derived from WJ of UC	Early Phase I	9 18 years and older	Early Consequence: Oxygen Saturation	NCT04456361
HB-AD-MSCs	Phase II, open label study, single-center trial	56 Child, Adult, Older Adult	Early Consequence: occurrence of hospitalization and signs related on SARS-CoV-2	NCT04349631
MSCs	Phase II, Interventional	20 18 years to 70 years	Early Consequence: evaluation of pneumonia recovery and adverse events	NCT04252118
UC-MSCs	Phase II, Multicenter, randomized-controlled trial, double-blind experiment	100 18 years to 75 years	Assessment of pneumonia Recovery	NCT04288102
UB-MSCs	Phase II RCT	20 30 years to 70 years	Evaluation of pneumonia and adverse effects	NCT04437823
Allogenic pooled olfactory mucosa-derived MSC	Phase I, Phase II	40 Age: 18 − 70	Number of infected persons treated	NCT04382547
UC-MSCs + Heparin along with best supportive treatment	Phase I/II double-blinded experiment	24 (18 years and older)	Harmlessness will be determined via the occurrence of intense side effects as measured via curing physician	NCT04355728
hUC-MSC (BX-U001) + supportive treatment	Phase1	9 18–80 years old	Assessment of safety	NCT03828344
MSCs in Inflammation-Resolution Programs of COVID-19 with ARDS	Prospective phase II trial	40 18 years and older	Recovery of lung damage score	NCT04377334
XCEL-UMC-BETA (WJ- MSC)	Phase 1, Phase 2, prospective, double-blind, randomized-controlled trial, parallel, placebo-controlled pilot	30 Age: 18 − 75	Number of infected persons who expired	NCT04390139
ACT-20-MSC	Phase 1, Phase II randomized-controlled trial, placebo-controlled trial	70 Age: 18–85	Death on day 30	NCT04398303
MultiStem	Phase II and III trial	400 Age: 18–89	Ventilator-Removal Days. Lack of side effects and tolerability	NCT04367077
MSCs-derived exosomes	Phase 1	24 18 Years to 75 years	Time to clinical recovery and safety	NCT04445220
UC-MSCs	Phase 2	16 Age: 18–80	Ventilation OI	NCT04269525
BM-MSCs	Phase I/II randomized-controlled trial	20 18 years to 75 years	Assessment of pneumonia recovery and harmlessness evaluation	NCT04346368
BM-Allo.MSC	Phase 1b randomized, double-blind, placebo-controlled study	45 Age: 18–80	Occurrence of AEs, death and its reason, and number of ventilator-removal days	NCT04397796

Di Trapani et al. showed that MSCs-Exos powerfully repressed the activities of B lymphocytes, contrasting with the actions of natural killer and T lymphocytes. Therefore, the immunological activity of MSC-Exos toward various cells of the immune system is associated with the kind of host cell, cellular maturation, cellular situation, and kind of diseases, among other agents. However, several investigations showed that MSCs-Exos could inhibit immune system cell generation and help an immunotolerant microenvironment [117].

Another study showed that a total of 11 patients detected with COVID-19-related ARDS who have been hospitalized in the ICUs of 2 hospitals enrolled in this trial. The patients were severely sick with acute hypoxemia and needed ventilation support. The patients were given three IV injections (200 × 10^6^ cells) every other day for a total of 600 × 10^6^ human UC-MSCs (for 6 patients) or placental MSCs (PL-MSCs; for 5 patients). There were no significant side effects diagnosed 24–48 h afterward from the MSCs injections. In addition, considerable decreases in serum amounts of TNF-α, IL-8, and CRP were detected in a total of 6 improved cases. In 5 patients, IL-6 levels were reduced, and IFN-α levels were reduced in 4 patients. Four patients who had symptoms of multiple organ dysfunction syndromes (MODS) or sepsis died 5–19 days (ordinary: 10 days) after the primary MSCs injection. Every six recovered patients were healthy, with no grievances of shortness of breath on day 60 after injection [118].

Shi et al. implemented a phase II trial to evaluate the effectiveness and safety of hUC-MSCs in treating acute SARS-CoV-2 with lung injury. COVID-19 patients were accidentally determined at a two-to-one ratio to administrate either UC-MSCs (40 × 10^6^ cells for each injection) or placebo on days 0, 3, and 6. In total, 100 SARS-CoV-2 patients were finally administrated with either UC-MSCs (65 participants) or a placebo (35 participants). Compared with the placebo, the UC-MSCs injection applied numerical recovery in total lung injury volume from the first day to the twenty-eighth day. These effects showed that UC-MSCs therapy is a safe and potentially efficient therapeutic method for COVID-19 with lung injury [119].

This investigation is a clinical pilot study that applies menstrual blood-obtained MSCs for the therapy of two acute COVID-19 patients with ARDS. MSCs were provided from menstrual blood attained from a healthy woman donor. MSC administration improves the immune indicators, such as helper T cells and lymphocytes, and reduces the inflammation indicators, including IL-6 and CRP. With MSC administration, the fraction of inspired O2 (FiO2) of the two COVID-19 patients slowly reduced, while the oxygen saturation (SaO2) and partial pressure of oxygen (PO2) were enhanced [120].

In another investigation, the effect of allogeneic BM-MSC-Exos in the therapy of acute COVID-19 was assessed in 24 patients with moderate-to-acute ARDS. A single infusion of 15 mL was injected into the enrolled cases intravenously, and the efficacy was checked from days 1 to 14 after the Exos infusion. A survival rate of 83 percent was detected, and out of 24 COVID-19 patients, 17 improved, 3 remained seriously ill (although constant), and 4 died because of unassociated therapy [121].

A phase 1 clinical study, called the START study, performed by Wilson et al. contained 9 COVID-19 patients with moderate-to-acute ARDS. MSCs were injected at low (1 × 10^6^ cells/kg), average (5 × 10^6^ cells/kg), and high (10 × 10^6^ cells/kg) for each three patients. The outcomes displayed that BM-MSCs were good tolerated in 9 patients with no therapy-associated side effects. In another investigation, Matthay et al. recruited 60 COVID-19 patients with moderate-to-acute ARDS. Forty patients received the highest amount of 10 × 10^6^ cells/kg according to the experiment of the previous phase I study, while the rest 20 patients received a placebo. The results showed that although disease intensity scores in the MSC group were higher than in the control group at baseline, death rates at 28 and 60 days did not differ significantly between the two groups. Furthermore, no MSC-related side effects were detected within 6 h of the injection in 40 patients with moderate-to-severe ARDS [122].

Aranzasti et al. (NCT04366271), McAuley et al. (NCT03042143), and Wang et al. (NCT04269525) are going to evaluate the remedial efficiency of UC-MSCs in the therapy of SARS-CoV-2-associated ARDS. The trial conducted by Aranzasti et al. is presently enrolling 106 patients who will be randomly isolated into the UC-MSC-under treatment and care-treated groups. UC-MSC-remediated patients will be injected with one dose of UC-MSCs. McAuley and colleagues will enroll 75 COVID-19 patients who will either administrate UC-MSCs or placebos (Plasma-Lyte). In a clinical trial Wang et al., (NCT04269525), COVID-19 patients were given UC-MSCs (3:3 × 10^7^ cells/50 ml/bag, 3 bags each time) [123].

Researchers used an injection of UC-MSC (1 × 10^6^ cells per kilogram) with convalescent plasma therapy (CP) in COVID-19 patients. The total lymphocyte amount was enhanced the next day and altered to normal on the fourth day. After IV injection of MSCs, reciprocal penetration was absorbed, and the pulmonary act was considerably recovered. Pre- and post-treatment with UC-MSCs, the COVID-19 patient's blood cell amount, IL-6, oxygenation index (OI), and partial pressure of oxygen observed a continuous reduction in total neutrophils and IL-6; lymphocyte amount, the OI, and partial pressure of oxygen slowly increased. Research laboratory information showed that UC-MSCs control the immune reaction, prevent the happening of hypercytokinemia, and repair the lung [124].

Häberle et al. treated five out of twenty-three acute COVID-19 patients with ARDS with an injection of MSCs. 1 × 10^6^ cells/kg was injected for 30 min, and the procedure was repeated in 3 COVID-19 patients twofold and in 2 patients 3 times. As a result, MSC injection was safe and well tolerated. The MSC group had a remarkably higher Horovitz score on discharge than the control cases [125].

### Advantages of MSC-based therapy

(1) Availability in separation from several tissues, including BM (most preferable source), peripheral blood, AD tissues, oral tissues, and menstrual blood (Men). MSCs can be isolated effectively from neonatal birth-related tissues, such as PL, UC (WJ and CB), and amniotic membrane (AM)/fluid (AF). In addition, after being isolated, MSCs can be preserved for subsequent interventions. (2) MSCs are multipotent SCs that can self-renew by dividing and can differentiate into several specialized cells; (3) MSCs can quickly develop to large volumes in a somewhat shorter term and can be deposited for repetitious therapeutic interventions; (4) until now, MSC-based clinical studies of MSCs did not demonstrate any severe adverse body responses to allogeneic MSCs; and (5) various clinical trial studies showed harmlessness of MSCs [126].

### Challenges to the use of MSCs in COVID-19 patients

Because the use of MSCs in COVID-19 therapy is classified as SC treatment method, thus the instructions of the International Society for Stem Cell Research (ISSCR) must be strictly followed. Furthermore, the ISSCR published “The Guidelines for the Clinical Translation of Stem Cells,” which includes the current criteria for advancing SCs-based treatments, resulting in proven curative capability for the patient. To advance the therapeutic result for the patients, MSC-based treatments must solve problems, such as expansion term, needed cell amount-dose, cell culture, and cell exposure to animal-isolated productions, which can influence the safety and efficiency importantly. Furthermore, long-term in vitro cultured SCs may lose their essential activation characteristics, including stemness and plasticity, or, even more, could undergo a malignant tumorigenic alteration [127].

Another problem in using MSCs in COVID-19 is the patients' eligibility for cell-based treatments. There is inadequate data on applying the MSC treatment method in patients with a history of chronic diseases or except situations, including cancer, autoimmune diseases, allergic diseases, gestation, and breastfeeding mothers. Another challenge is that many clinical studies applying MSCs or derived Exos in the therapy of COVID-19 are in phase I/II, which resulted in unsatisfactory results. Clinical injection of MSCs needs potent proof and verification of safety and effectiveness, which is attained via assessing likely adverse events and unwanted long-term consequences [111].

Though BM is the most popular origin for separating MSCs, the harvesting process is invasive, and the cell counts are confined. Furthermore, ARDS influences the immunomodulatory efficacy of BM-MSCs and disrupts their possible usage for autologous transplantation. Through various investigations have assessed the curative efficacy of MSCs from other origins in ARDS, it is not yet clear which one can provide the best curative efficacy. Cell injection amounts are essential for the clinical usage of MSC treatment. In investigational patterns, the amount of MSCs injected at a once dosage ranges from 5 × 10^4^ to 3.6 × 10^7^ cells. Clinically, this range in a 25-g mouse would correspond to 2 × 10^6^ to 1.44 × 10^9^ cells/kg in humans. Such amounts are faced with technological and usable problems. Up to now, 1 × 10^7^ cells/kg is the maximum dosage applied in clinical trials [128].

## Conclusion

In this review, the nature of the inflammatory responses that have been recognized in infected people with COVID-19 was discussed. Besides, several preliminary results that confirm the emergence of MSCs as a promising therapeutic candidate to manage this infection were outlined. The usage of MSCs for ARDS associated with COVID-19 is highly experimental. COVID-19 patients with severe pneumonia reported amelioration of respiratory function due to MSC infusion due to yet unclear modulation of hypercytokinemia and protection of epithelial and endothelial cells. The safety of MSCs, the vast preclinical effectiveness information of MSCs therapy, and several positive reports of clinical trials of MSCs therapy in COVID-19 patients have shown the beneficial therapeutic potential of MSCs for SARS-CoV-2 patients with critical manifestations and hyper-inflammatory states. However, it was noted that MSCs should not be considered a cure-all for SARS-CoV-2, and the injection of MSCs should not happen in the lack of severe problems of the disease such as ARDS and hypoxic respiratory failure.

## Data Availability

Not applicable.

## Abbreviations

MSCs: Mesenchymal stem cells
SARS-CoV-2: Severe acute respiratory syndrome coronavirus 2
ACE2: Angiotensin-converting enzyme 2
ALI: Acute lung injury
ARDS: Acute respiratory distress syndrome
RBD: Receptor-binding domain
6-HB: 6-Helix bundle
MHC: Major histocompatibility complex
APCs: Antigen-presenting cells
PBMCs: Peripheral blood mononuclear cells
